# Supplementary material for: A Gs-coupled purinergic receptor boosts Ca2+ influx and vascular contractility during diabetic hyperglycemia
Source: eLife. 2019 Mar 1;8:e42214. doi: 10.7554/eLife.42214 (PMC6397001; doi:10.7554/eLife.42214)
Supplement: Supplementary file 1. [file elife-42214-supp1.docx]

**Supplementary file 1: K^+^-induced constriction and baseline and passive diameters of arteries from wild type and S1928A mice**

|  | **peak 60 mmol/L K^+^ constriction (%)** | **baseline diameter (μm)** | **passive diameter (μm)** |
| --- | --- | --- | --- |
| Figure 1A (20 mM D-glu) | 65 ± 5 | 108 ± 7 | 143 ± 7 |
| Figure 1A (20 mM D-glu + apyrase) | 73 ± 4 | 109 ± 5 | 138 ± 3 |
| Figure 1 – figure supplement 1B (20 mM mannitol) | 61 ± 5 | 112 ± 9 | 137 ± 8 |
| Figure 6G (20 mM D-glu + NF340) | 77 ± 7 | 109 ± 8 | 142 ± 5 |
| Figure 6I (20 mM D-glu + MRS2578) | 65 ± 3 | 135 ± 5 | 166 ± 3 |
| Figure 6 – figure supplement 1D (MRS2179 + 20 mM D-glu + NF546) | 72 ± 4 | 115 ± 12 | 137 ± 10 |
| Figure 6 – figure supplement 1E (20 mM D-glu + MRS2179) | 57 ± 3 | 126 ± 2 | 149 ± 4 |
| Figure 7E (wt NF546) | 60 ± 9 | 124 ± 4 | 145 ± 4 |
| Figure 7F (20 mM D-glu + NF546) | 86 ± 7 | 121 ± 7 | 145 ± 6 |
| Figure 7F (S1928A NF546) | 63 ± 8 | 108 ± 15 | 132 ± 15 |

Values are mean ± SEM.
