# Supplementary material for: A Gs-coupled purinergic receptor boosts Ca2+ influx and vascular contractility during diabetic hyperglycemia
Source: eLife. 2019 Mar 1;8:e42214. doi: 10.7554/eLife.42214 (PMC6397001; doi:10.7554/eLife.42214)
Supplement: Supplementary file 2. [file elife-42214-supp2.docx]

**Supplementary file 2: Arterial tone from wild type and S1928A mouse arteries**

| **treatment** | **D-glucose (mM)** | **arterial tone (%)** |
| --- | --- | --- |
| control | 10 | 24 ± 3 |
|  | 20 | 34 ± 5* |
| apyrase | 10 | 21 ± 2 |
|  | 20 | 20 ± 3 |
| mannitol | 10 | 19 ± 3 |
|  | 20 mannitol | 19 ±3 |
| NF340 | 10 (-) | 23 ± 6 |
|  | 10 (+) | 25 ± 6 |
|  | 20 (+) | 24 ± 6 |
| MRS2578 | 10 | 13 ± 1 |
|  | 20 | 20 ± 1* |
| MRS2179 | 10 (-) | 17 ± 4 |
|  | 10 (+) | 18 ± 4 |
|  | 20 (+) | 30 ± 5^‡^ |
|  | 20 (+) + NF546 | 30 ± 5^‡^ |
| MRS2179 | 10 (-) | 15 ± 2 |
|  | 20 (-) | 26 ± 2^‡^ |
|  | 20 (+) | 26 ± 2^‡^ |
| NF546 (wt) | 10 (-) | 14 ± 2 |
|  | 10 (+) | 21 ± 3* |
| NF546 | 10 (-) | 17 ± 4 |
|  | 20 (-) | 31 ± 3^‡^ |
|  | 20 (+) | 31 ± 4^‡^ |
| NF546 (S1928A) | 10 (-) | 19 ± 3 |
|  | 10 (+) | 18 ± 3 |

Values are mean ± SEM (**P* < 0.05, Wilcoxon matched pair test); ^‡^*P* < 0.05, Friedman with Dunn’s multiple comparisons). (-) indicates exclusion of treatment, (+) indicates exposure to treatment.
