# Supplementary material for: A Gs-coupled purinergic receptor boosts Ca2+ influx and vascular contractility during diabetic hyperglycemia
Source: eLife. 2019 Mar 1;8:e42214. doi: 10.7554/eLife.42214 (PMC6397001; doi:10.7554/eLife.42214)
Supplement: Supplementary file 3. [file elife-42214-supp3.docx]

**Supplementary file 3: Human nondiabetic patients undergoing surgical sleeve gastrectomy**

| **age (years)** | **gender** |
| --- | --- |
| 44 ± 2 | 34 F / 3 M |
